# Field Phenotyping Strategies and Breeding for Adaptation of Rice to Drought^†^

**DOI:** 10.3389/fphys.2012.00282

**Published:** 2012-07-24

**Authors:** Ken S. Fischer, Shu Fukai, Arvind Kumar, Hei Leung, Boonrat Jongdee

**Affiliations:** ^1^School of Agriculture and Food Science, University of QueenslandBrisbane, QLD, Australia; ^2^International Rice Research InstituteLos Baños, Laguna, Philippines; ^3^Ubon Rice Research CenterUbon Ratchathani, Thailand

**Keywords:** drought adaptation, drought resistance traits, phenotyping, target population of environments, Water stress

## Abstract

This paper is a section of the book “Drought phenotyping in crops: from theory to practice” (Monneveux Philippe and Ribaut Jean-Marcel eds, published by CGIAR Generation Challenge Programme. Texcoco, Mexico). The section describes recent experience in drought phenotyping in rice which is one of the most drought-susceptible crops. The section contains genetic and genomic resources for drought adaptation and methods for selection of drought-resistant varieties in rice. In appendix, there is experience from Thailand on integration of direct selection for grain yield and physiological traits to confer drought resistance.

## Challenges and General Information

### Importance of rice in the human diet

Rice is the staple food for approximately 340 million poor people in South Asia and 140 million each in Southeast Asia and sub-Saharan Africa (International Rice Research Institute; IRRI, 2006). It is the basic food crop of Asia, providing over 30% of the calories consumed in the region. Overall, there is an estimated global need for an additional 116 million tons of rice by 2035 as compared to 439 million tons production in 2010 (Seck et al., 2012). The estimated annual increase is expected to be 13% for the first 10 years and 12% in the next 15 years as population growth drops and people diversify from rice to other crops (Seck et al., 2012).

### Cultivated area and yield performance under optimal conditions

Irrigated rice accounts for almost 75% of total world rice production. It was the source of the large increases of productivity leading to the Green Revolution. However, technological progress in rice cultivation has slowed down substantially since the early 1990s from the 2.5% per year during the first two decades of the Green Revolution to about 1.1% per year since the late 1980s. The stagnation in yield growth is because yields are approaching the practical potential of the rice crop growing under favorable environments (IRRI, 2006). Further increases will have to come from new breakthroughs in increasing the yield potential under favorable conditions and from increased performance of rice growing under less favorable conditions. In both scenarios it is likely that there will be less water and probably less available labor. Thus, research needs to increase the productivity of water for both irrigated and rain-fed systems.

### Importance of drought in rice farming

Rain-fed rice ecosystems are home to 80 million farmers on 60 million ha. Progress has been slow in improving productivity, and drought is a major constraint affecting rice production, especially in rain-fed areas across Asia and sub-Saharan Africa. Pandey et al. (2007) estimate that at least 23 million ha of rain-fed rice area (20% of the total rice area) in Asia are drought-prone. Even in traditionally irrigated areas, which account for almost 75% of total rice production, drought is becoming an increasing problem because of water scarcity resulting from rising demand for water for competing uses. Drought imposes a serious economic burden on society and has been historically associated with food shortages of varying intensities, including those that have resulted in major famines in different parts of Asia and Africa. For example, Pandey et al. (2007) estimate production losses of 36% of the average value of production in eastern India in drought years. This represents a massive loss of US$856 million and, on a yearly basis, a loss of 6.8% of the average value of output in India.^1^In addition to the direct effects on production, there are indirect effects of drought which may be felt over several years. Its impact can even span generations as, e.g., when children fail to recoup lost educational opportunities (Pandey et al., 2007).

### Opportunities to improve drought tolerance in rice

O’Toole (2004) suggests that the probability of success in developing drought-tolerant varieties of rice^2^ is enhanced because of scientific progress in: (i) understanding the physiological mechanisms that impart tolerance of drought in rice (Fukai and Cooper, 1995); (ii) new molecular tools; and (iii) the practical application of this knowledge and tools for screening selection and improvement of rice germplasm for drought (Atlin, 2003; Jongdee et al., 2006; Lafitte et al., 2006; Bernier et al., 2007; Venuprasad et al., 2007a). Also several international workshops and training courses have dealt with the theory and practice of science-based screening of rice for drought tolerance (Ito et al., 1999; IRRI, 2002; Saxena and O’Toole, 2002; also see: www.plantstress.com). Bennett (2003) has provided an overview of the opportunities for increasing water productivity of major food crops through plant breeding and molecular biology, and Fischer et al. (2003a) have provided a practical manual with updated information for rice breeders regarding the theory and practice of breeding for drought tolerance in rice.

In recent years, rice research programs in India (Babu et al., 2003), China (Zheng et al., 2004, Thailand (Jongdee et al., 2006), Laos and Cambodia (Report to Rockefeller Foundation, 2006), The Philippines (Atlin, 2003; Lafitte et al., 2006; Bernier et al., 2009), and Brazil (da Silveira Pinheiro, 2003) are now selecting for drought tolerance as a specific trait to improve performance under rain-fed conditions. O’Toole (2004) identifies two innovations that characterize this new and successful approach. First, the work of physiologists, geneticists, and breeders led to more reliable control of water-stress severity and duration at the critical yield determining growth stages, and this gave rise to the development and utilization of effective selection measures. Second, by employing farmers’ participatory selection groups as the final evaluators (Witcombe et al., 2002), real and lasting progress is now within reach. While end-user evaluations are important to any breeding program, they are particularly critical across drought-prone regions, where local variation in soils and landscape result in strong genotype-by-environment interactions (GEI). O’Toole (2004) further suggests that these innovations, when taken together, bode well for the large-scale dissemination of new drought-tolerant rice varieties across Asia in the very near future.

## Relevant Research Available

### Genetic and genomic resources

Since genetic resources are being produced continuously in breeding and genetic research programs around the world, it is not possible to provide an exhaustive list of current genetic stocks. Instead, it is more meaningful to indicate the principles and approaches behind the development and use of genetic resources relevant to drought breeding, and illustrate each principle with specific examples.

Due to the complexity of genetic control, genetic stocks for drought research require unique features that enable detection of not only individual genes but also possibly complex genetic loci (e.g., gene clusters or interacting loci). Table 1 summarizes the categories of publicly accessible genetic resources useful for drought research and breeding.

**Table 1:** **Overview of rice genetic resources useful for drought research and breeding**.

Specialized genetic stock	Feature	Produced and accessible at
Chromosomal segment substitution lines (CSSL)	Two *sativa* × *glabberima* libraries Four wild interspecific libraries using different wild rice relatives Four *japonica* × *indica* libraries	Centro Internacional de Agricultura Tropical; International Center for Tropical Agriculture (CIAT) National Institute of Agrobiological Sciences (NIAS), Japan
Recombinant inbred lines [tolerant × sensitive, including doubled haploid (DH) lines]	Approximately six commonly used populations	IRRI, Institut de recherche pour le développement (IRD), France
Breeding populations	Advanced lowland and upland breeding lines, new rice for Africa (NERICA) series	IRRI, WARDA (Africa Rice Center)
Introgression lines	*glabberima* × *sativa*	CIAT, IRRI, WARDA
Near-isogenic lines	Derived from breeding populations evaluated under field conditions	IRRI
Mutants	Insertions/activations/deletions	Multiple institutions with different degrees of accessibility. Materials can be requested from individual institutions through Standard Material Transfer Agreements

*Source: Information assembled from discussion sessions at the Third Annual Research Meeting of the Generation Challenge Programme (GCP), 16 September 2007, Johannesburg, South Africa*.

Specialized genetic stocks can be classified broadly as those derived from natural genetic variation, and those induced by artificial means. This distinction is useful in a practical sense because different genetic and molecular approaches are required to analyze the materials. A wide range of natural variation is harbored in the deep genepool of rice germplasm comprising domesticated and wild species (Leung et al., 2007). This genepool represents genetic diversity resulting from thousands of years of natural selection and more recent selection through breeding. Thus, the genetic variation present in germplasm is likely to be agronomically relevant. On the other hand, artificially induced variation is generated by randomly mutating the genome to increase the probability of detecting novel variation, or by over-expressing or silencing specific genes. Mutants offer the advantage of carrying precise genetic alterations in the genome, and are, therefore, ideal for investigating genes with major phenotypic effects. Being essentially isogenic, mutants are useful for examining genetic loci with quantitative effects, provided that fixed lines are evaluated in replicated trials. A limitation of mutation analysis is that it is confined to analysis of two alternate alleles in a fixed genetic background. Thus, for phenotypes that are conditioned by large gene blocks or complex genetic interactions, mutation analysis alone is not adequate and should be complemented with analysis of natural diversity present in the germplasm.

### Random and targeted induced variation

#### Randomly induced mutations

To take advantage of rice genome sequence information, a large collection of rice mutants has been produced in the scientific community (Hirochika et al., 2004). These mutants can be classified broadly as transgenic and non-transgenic. The transgenic mutants are produced by transformation vectors, primarily transfer DNA (T-DNA)-based vectors. Depending on the features of the vectors, insertion events can cause knockout or activation mutations. Activation mutations are unique in that a normally “dormant” gene can be activated to unleash novel variation (Leung and An, 2004).

A main advantage of insertion mutants is that the insertion sites can be sequenced, producing a large dataset of flanking sequence tags (FST; for articles on such specialized mutant populations produced in Asia see An et al., 2007). With the current FST databases, there is more than a 60% probability of finding a mutation in a given gene, providing a reverse genetics tool to search for knockout or activation mutants in genes suspected to play a role in response to water stress. An ongoing project of the Generation Challenge Programme (GCP) is to exploit this FST database to identify and phenotype mutants with insertions in stress-associated genes (SAGs; A. Pereira, personal communication).

Non-transgenic mutants include those produced by conventional chemical and irradiation mutagenesis. Wu et al. (2005) described a large collection of *indica* rice mutants produced by fast neutron, gamma ray, ethyl methanesulfonate (EMS), and diepoxybutane. ^3^For forward genetics screening of drought tolerance, non-transgenic mutants are advantageous because they can be freely distributed and tested under field conditions. With very few exceptions, it remains difficult to conduct extensive field screening of transgenic mutants. Not all insertion mutants are transgenic. For example, the Tos17 population caused by insertions of a retrotransposon element can be evaluated under field conditions.

#### Targeted silencing and activation of specific genes

For genes with hypothesized function in drought tolerance, there is the option of creating “down-expression” mutants by silencing the gene by the RNA interference (RNAi) technique or more recently by artificial micro-RNA (Warthmann et al., 2008). By combining over- and under-expression, the function of the gene can be inferred conclusively if side effects of the introduced RNAi construct can be excluded. Recent examples include the functional characterization of the SHINE and HARDY genes (Karaba et al., 2007). Expression of SHINE and HARDY are reported to confer water-use efficiency (WUE) in rice, although their phenotypic effects have not been evaluated under field conditions.

It is hypothesized that ERECTA is a “master” gene regulating transpiration efficiency in *Arabidopsis* (Masle et al., 2003). Mutations in ERECTA have been found in the Tos17 population and in Pohang collection (Hirochika et al., 2004). However, attempts to phenotype the ERECTA mutants have proved difficult because of the extensive somaclonal variation expressed by the mutants derived from tissue culture. Most of the transgenic mutants are maintained in early generations (T_1_ or at most T_2_) and some of them continue to segregate in characters unrelated to the disrupted gene. It is important that mutants be backcrossed to the wild type to clean up the background mutations before extensive phenotyping (Dworkin et al., 2009).

### Capturing natural variation through specialized genetic stocks

#### Mapping populations

Mapping populations can broadly be defined as genetic populations that can be used to demonstrate inheritance of traits. In general, such a population is derived from a cross between two genetically distinct parents. Taking this broad definition, a large collection of rice genetic stocks are available for defining inheritance of drought response (see Table 2).

**Table 2:** **Segregating populations generated by drought breeding programs for genetic analysis and breeding for drought tolerance; genetic stocks maintained at IRRI (Source: information provided by Arvind Kumar, IRRI)**.

Population name	Parent A	Parent B	Type	Population size
IR78875	Apo	IR64	RIL	200
IR78877	Apo	IR72	RIL	200
IR78908	Vandana	IR64	RIL	200
IR78910	Vandana	IR72	RIL	200
IR79971	Vandana	Way Rarem	RIL	500
IR78937	IR 47701-6-B-1	IR55435-05	RIL	500
IR79913	IR 55419-04	Way Rarem	RIL	500
IR79915	IRRI 132	IR55419-04	RIL	500
IR72757	Bala	IR64	RIL	400
IR79971	Vandana	Way Rarem	RIL	500
IR80508	IRRI 132	AUS 257	RIL	500
IR81023	IRRI 143	CT 6510-24-1-2	RIL	500
IR81027	IRRI 143	UPLRI 7	RIL	500
IR81047	IR 01A102	CT 6510-24-1-2	RIL	500
IR81063	NOK	IR74371-46-1-1	RIL	500
IR81896	Apo	Swarna*2	BC^a^	500
IR81895	Apo	Mahsuri*2	BC	200
IR84179	IR 78877-208-B-1-2	IR72*2	BC	500
IR84182	IR 78878-53-2-2-2	IR 72875-94-3-3-2*2	BC	400
IR83632	IR 78910-34-B-2-2	IR72	RIL	500
IR84184	IR 78908-63-B-1	IR64*2	RIL	300
IR83614	IR78875-131-B-1-2	IR64	RIL	800
IR84148	IR79971-B-55-B-B	Way Rarem	RIL	500
IR83575	IR 79913-B-102-B-5	Way Rarem	RIL	200
IR81024	IR77298-5-6	IR71525-19-1-1	RIL	500
IR84129	IR77298-5-6	IR77298-14-1-2	RIL	500
IR83641	IR77298-14-1-2	IR64	RIL	300

*^a^BC, backcross*.

In rice, the most common mapping populations are recombinant inbred lines (RILs) derived from two parental lines with high and low traits for drought tolerance. A key advantage of ‘s is that they can be “immortalized” (Collard et al., 2005) as advanced F_7_ generations or beyond. The population can be evaluated repeatedly over time and over locations to generate a large amount of phenotype data. Historically, RIL mapping populations are made to map component or secondary traits contributing to drought response. For example, the well-studied IR64 × Azucena RIL or double haploid populations have been used for mapping osmotic adjustment among other traits. However, using these quantitative trait loci (QTLs) to reconstitute drought-tolerant varieties has had limited success (Venuprasad et al., 2009).

More recently, considerable effort has been devoted to extracting segregating materials directly from breeding programs and converting them into advanced genetic stocks that can serve the dual purposes of QTL/gene identification and breeding. A main advantage of these materials is that they are selected for yield under stress in field conditions. Hence, the traits or QTLs under investigation have a high probability of being relevant agronomically. Examples of advanced breeding populations for detecting QTLs for yield under drought stress are shown in Table 3.

**Table 3:** **Rice breeding populations for detecting large-effect QTLs for yield under drought stress under upland and rain-fed lowland production systems; genetic stocks maintained at IRRI (Source: information provided by Arvind Kumar, IRRI)**.

Population	Generation	Rice production system	Reference
Vandana × Way Rarem	F_3_ derived, BC_1_F_3_, BC_3_F_3_	Upland	Bernier et al. (2007)
IR55419-04/Way Rarem	F_3_ derived, BC_1_F_3_, BC_3_F_3_	Upland	IRRI, unpublished
Aday Sel/IR64	BC_3_F_5_, approaching NIL	Rain-fed lowland	Venuprasad et al. (2007b)
Apo × Swarna	F_3_ derived, BC_1_F_3_	Rain-fed lowland	IRRI, unpublished
CT9993-5-10-1-M/IR62266-42-6-2	DH	Rain-fed lowland	Kumar et al. (2007)

#### Near-isogenic lines

Near-isogenic lines (NILs) have a special place in genetic analysis and breeding. A pair of NILs with and without the target trait provides the best genetic materials to define unique chromosomal regions conditioning phenotypes, and eventually leads to gene cloning. Compared to disease resistance, NILs for drought tolerance are neither common nor well developed in rice. To fill this gap, advanced backcross lines have been developed using breeding lines with demonstrated field performance against drought stress (see Table 3). Their development can be facilitated through the use of heterogeneous inbred families (HIFs) resulting in an NIL that carries a heterozygous region for the target QTL. Such a line can be selfed to produce a pair of lines homozygous at the target region. Several pairs of NILs are now available for detecting the chromosomal regions conferring large effect for drought tolerance.

#### Multiparent advanced generation intercross populations

The multiparent advanced generation intercross (MAGIC) approach originally developed in animal genetics is now being explored in plants (Cavanagh et al., 2008). In this approach, recombinant populations are generated by intercrossing a number of selected founder lines (between 8 and 16 genotypes) that are genetically distant from each other and carry unique genetic attributes. The resulting populations are subjected to multiple cycles of intercrossing to maximize recombination between chromosomes. At an advanced stage, a large (>2,000) RIL population is established. This recombinagenic population is expected to exhibit novel variation and to provide a permanent resource for high-resolution mapping.

The International Rice Research Institute (IRRI) has initiated the development of MAGIC populations for rice. Two populations will be developed: one will be targeted at irrigated and one at rain-fed ecosystems that are relevant to both Asia and Africa, recognizing that the utility of the two populations will overlap. Each population will have eight founders, selected either as elite, well-adapted varieties for the respective environment, or as potential donors of useful germplasm not found within the current elite pool. Within 3 years, it is expected to have sufficient seeds from the MAGIC populations for a first round of phenotypic evaluation.

#### Diverse germplasm panel for association genetics

Genetic association analysis makes use of the fact that, within an unstructured genepool, blocks of chromosome can be found associated with certain phenotypes. Unlike conventional linkage analysis, association analysis exploits the large number of historical meioses (genetic recombination events) in the germplasm. The resolution of this association depends on the levels of linkage disequilibrium (LD).

Rice is particularly suitable for developing an association genetics platform for determining the relationship between chromosomal blocks and traits of interest. Under the OryzaSNP project coordinated by the International Rice Functional Genomics Consortium (IRFGC), there is now an extensive single nucleotide polymorphism (SNP) database consisting of over 150,000 SNPs across 20 diverse rice genotypes (McNally et al., 2006; OryzaSNP website^4^). This OryzaSNP dataset, together with other SNP data from the rice research community, provides the tools for high-resolution genotyping. The OryzaSNP consortium is mobilizing the community to conduct a comprehensive survey of genome-wide SNP variation in more than 2,000 diverse rice genotypes selected based on diversity, utility in breeding, and geographical representation. If successfully implemented, the SNP haplotype and phenotype database of a large collection of rice germplasm and breeding lines will provide a powerful platform for relating phenotypes to specific regions of chromosomes in rice.

In summary, genetic variation for conditioning drought tolerance exists in rice but such variation must be captured and displayed in a suitable genetic background amenable to genetic analysis and breeding manipulation. To understand and use this genetic variation for breeding, it is necessary to continue to invest in producing and maintaining well-managed, publicly accessible, high-quality genetic stocks relevant to drought research. Such genetic stocks should enable QTL mapping for drought tolerance at 1 cM (0.5–1 Mb) resolution and they should be useful donors in prebreeding. Learning from the experience of breeding for disease resistance, developing breeding-ready NILs with sequence-indexed chromosomes and known phenotypic contribution to drought tolerance should prove highly valuable to breeding for drought tolerance.

### Breeding strategy

Generally, breeding methods for rain-fed rice have been strongly influenced by experiences in irrigated rice, where the crop is usually grown under stress-free conditions and where yields in farmers’ fields approach those on experiment stations. Most conventional plant breeders in rain-fed systems use the early screening phase to select for traits such as height, maturity, plant type, pest tolerance, and grain quality, often under well-watered conditions on research stations. Only at the advanced testing stage, when relatively few genotypes remain, are entries evaluated under the stress conditions of farmers’ fields. The outcome is often a variety that performs well under well-watered conditions but poorly under stress.

In contrast to this conventional approach, growing evidence indicates that varieties developed for improved yield under drought stress will respond to well-watered conditions if there is early selection in both environments. There are several reasons for plant breeders’ apprehension about selection under drought stress. Uppermost among them is that the target environment where selection and testing work are done is often spatially variable in terms of rainfall. Because of the variability in the rain-fed environment, breeders are searching for more reliable phenotyping protocols that can accelerate progress. However, breeders must be aware that there is a “chain of correlation” between performance in a screening environment and performance in farmers’ fields. Thus, before embarking on a phenotyping protocol, the breeder must test the assumption that the performance in a given drought protocol is predictive of performance on-farm under farmer management.

Rain-fed rice is grown in two major ecosystems, rain-fed lowland where the rainwater is stored through “bunding” of the fields such that the crop is exposed to anaerobic and aerobic conditions, and upland rice where the crop grows under aerobic conditions. There is another emerging ecosystem of interest and that is the traditional irrigated rice system where there is increasing pressure on water availability. Rice researchers are developing “aerobic rice” for this emerging ecosystem. Of these, the former is by far the dominant and accounts for around half of the rice area worldwide. It is the main focus of this case study in breeding for drought resistance in rice.

### Plant water strategy

#### Background and simple model for yield under drought

**Figure 1:**
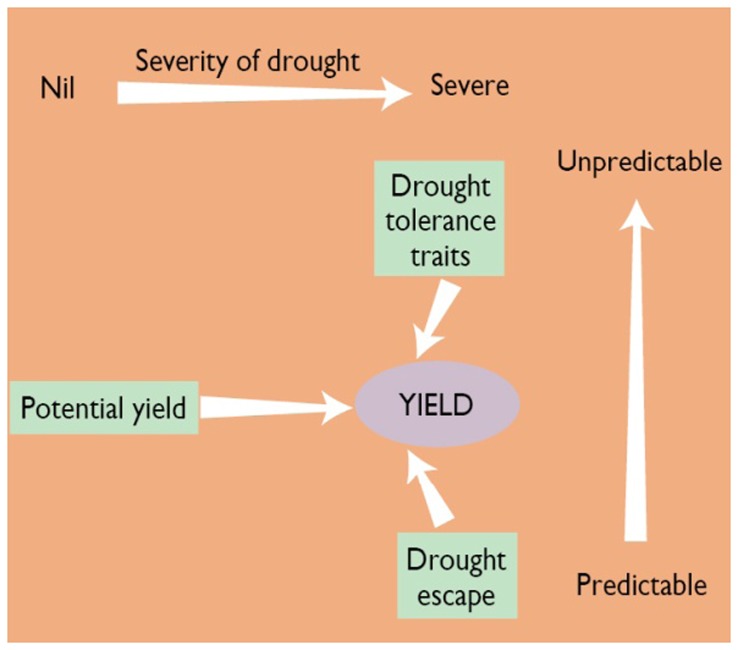
**Schematic diagram of three components of yield under drought-prone environments (potential yield, phenology, and drought-tolerance traits) and yield relationship in different types of drought in rain-fed rice**. Note that when drought is not present, yield potential determines grain production. Moving to the right in the figure, drought becomes more severe and drought escape or drought tolerance becomes more important. The vertical axis represents the predictability of drought. If drought is very predictable (bottom), drought escape through changing phenology or planting date is a good option. As drought becomes more unpredictable (moving up on the axis), drought-tolerance traits become more necessary (Redrawn from: Fukai and Cooper, 2001).

Numerous workers have studied the complex processes, mechanisms, and traits that determine rice yield under moisture-limiting conditions. Fukai and Cooper (2001) have summarized this complexity, and focus on three broad mechanisms that influence yield depending on the severity and predictability of the drought in the TPE where the crop is grown (Figure 1). The contribution of phenology to escape from predictable drought is well understood. Its role in unpredictable drought occurring around flowering is still under investigation. There is considerable evidence that yield potential contributes to yield under drought, with recent evidence from the work of Kumar et al. (2007) showing a genetic correlation of 0.8 between yield under stress and non-stress. This indicates that much of the yield under drought is accounted for by yield potential. Plant breeders have improved yield potential, mainly by increasing harvest index (HI) through shorter plants and earlier flowering with more tillers and greater spikelet number, and, to a lesser extent, green leaf duration (GLD), by maintaining a larger leaf area for a longer period.

The main approach for breeding for drought-prone environments is to: (i) improve yield potential and, depending on the type of drought, select for the appropriate combination of maturity to avoid stress during the reproductive stage; and (ii) select for tolerance to drought stress during the reproductive period, and avoid plant types that use a lot of water prior to flowering (i.e., produce large amounts of dry matter (DM) and run out of water at the critical stage of flowering. In upland rice, as in other aerobic crops, there may also be opportunities to increase the amount of water transpired through more vigorous root systems.

#### Putative traits for drought tolerance

There are many putative traits that have been studied for their use in breeding for drought tolerance in rice, as listed in Table 4. However only a few can be recommended for use in a practical breeding program at this time. They are described in detail later. Research continues on some of the putative traits but at this stage they are not recommended for application in a breeding program.

**Table 4:** **Putative traits for drought tolerance**.

Trait	Proposed function	Comments	Reference
Leaf-rolling score	To reduce transpiration	Used during vegetative stress; high heritability (ca. 0.8), but low/no association with yield. Good as an indicator of stress in an experiment	Courtois et al. (2000)
Osmotic adjustment (OA)	To allow turgor maintenance at low plant water potential	*Indica* types have high OA, *japonica* types have low OA. This trait has been associated with a yield advantage in wheat, especially in terminal stress environments	Lilley et al. (1996)
Deeper, thicker roots	To explore a greater soil volume	There is evidence from MAS that increasing root mass below 30 cm results in greater yield under stress. No evidence on root thickness *per se*. Large-scale screening is difficult	Yadav et al. (1997)
Root-pulling resistance	For root penetration into deeper soil layers	Is correlated with larger root system	Pantuwan et al. (2002b)
Greater root penetration ability	To explore a larger soil volume	Most studies use artificial barriers with known mechanical resistance. There is some controversy regarding how well this mimics the soil situation	Ali et al. (2000), Clark et al. (2000)
Membrane stability	To allow leaves to continue functioning at high temperature	Genotypic differences are clear. Has been linked to heat tolerance in several species. Link to drought tolerance is less evident	Tripathy et al. (2000)
Leaf relative water content (RWC)	Indicates maintenance of favorable plant water status	Trait has rather low heritability; QTLs not repeatable	Courtois et al. (2000)
Water-use efficiency (WUE)	Indicates greater carbon gain per unit of water lost by transpiration	Carbon isotope discrimination (Δ^13^C) provides an integrated measure of WUE over the season. It has been used successfully for crops in more arid climates but has not been applied to rice	Specht et al. (2001)

*Note that QTLs have been identified for these secondary traits. Now they need to be tested for their relationship with performance under drought stress, and suitable high-throughput screening strategies must be developed (Source: Lafitte et al., 2003)*.

### Phenotyping traits^5^

#### Why use secondary traits?

Grain yield under stress is the primary trait for selection in breeding programs for drought-prone environments. However, it is sometimes useful to screen for secondary traits as well. These traits are plant characteristics that are associated with yield under stress, and they can provide additional information for breeders to use when they make selections. Breeders who select for disease scores, plant height, and flowering date are all using secondary traits. For a secondary trait to be useful in a breeding program, it has to pass five tests:

• It must be genetically correlated with grain yield in the predominant stress situations that occur in the target environment.
• It should not be affected very much by environment; that is, it should be highly heritable in the screening system used.
• There must be variation among lines for the trait.
• It should not be associated with poor yields in the unstressed environment.
• It must be possible to measure the trait rapidly and economically.

#### When to use secondary traits?

Secondary traits can improve the selection response if they contribute in one of the following ways:

• They improve precision *if* the heritability of yield is reduced by stress and the heritability of the secondary trait is not reduced by stress.
• They facilitate manipulation of the drought environment. It may be easier to reveal variation in the secondary trait than to reveal variation in yield. For example, the timing of stress has a very large effect on the extent to which yield is reduced, so it is hard to compare lines with different flowering dates. If a secondary trait is less sensitive to the growth stage of the crop, this makes it easier to compare lines of different maturity.
• They focus the selection on a specific type of drought, yield being the summation of all stresses, including those not directly associated with water.
• They are cheaper and easier to measure than grain yield under stress. Frequently, experiments are lost because of pests or weather damage before the final yield can be recorded. In such cases, a good secondary trait allows useful data to be collected from the experiment.

## Methodology and Field Trials

The following provides some practical advice for breeding rice for drought-prone environments with a focus on practical protocols for phenotyping. The focus is on rain-fed lowland rice. The material is taken from Fischer et al. (2003a).

### Trial planning

#### Definition of the target environment^6^

There is no one environment, even on the same farm, for which a breeding program is targeting improvement toward. Rather, there are several environments that will change from year-to-year and from field-to-field. These are referred as “the target population of environments” (TPE; Cooper et al., 1997). Each breeding program must clearly define the TPE for which it is developing varieties. Thus, a TPE is the set of all environments, fields, and seasons in which improved varieties are expected to do well. However, the environments must be sufficiently similar for one genotype to perform well in all of them.

#### How to determine the target population of environments for rain-fed lowland rice

Start with spatial information on water availability at the sub-ecosystem level. A commonly used system for characterizing rain-fed lowland systems is that of sub-ecosystems defined by Khush (1984), and later modified by Mackill et al. (1996). Three of these sub-ecosystems are relevant to breeding for drought tolerance:

• rain-fed, shallow, favorable sub-ecosystem, where rainfall and water control are generally adequate for crop growth, and only short periods of drought stress or mild submergence occur
• rain-fed, shallow, drought-prone sub-ecosystem, with either a short rainy season or a long and bimodal rainy period
• rain-fed, shallow, drought- and submergence-prone sub-ecosystem, where drought and submergence may occur within the same growing season or in different seasons.

#### Use the knowledge and experience of farmers and breeders to characterize local environments

Farmers, agronomists, and breeders who are familiar with a field and have observed rice crops grown in it over several years can usually determine the type of drought risk it is subject to quickly and accurately. This is largely a function of toposequence position and soil texture. Upper terraces, particularly those with light soils, are most subject to drought risk. Using the knowledge of experienced farmers and researchers is the most accurate and simple approach for assigning fields to a particular TPE. As a general rule, drought risk is most severe in entirely rain-fed upper fields, in which standing water rarely accumulates, and in which farmers grow short-duration, photoperiod-insensitive varieties.

#### Use the performance of known varieties to define the target population of environments

Most breeding programs routinely collect data from variety trials grown over all environments, called multienvironment trials (METs). These historical data can be reanalyzed using the statistical package CROPSTAT^8^ to determine the clustering or grouping of environments, based on the correlation of variety means across trials. The results can be used to define the TPE. There is a simple way to group locations and fields into the TPE, using the correlation of variety means from trials testing the same set of varieties. The repeatability (also known as the broad-sense heritability or H) of a three- or four-replicate trial usually ranges from 0.3 to 0.4. This is also the expected correlation of variety means in trials conducted in different fields if there is not much GEI between them. Thus, if the correlation between cultivar means in trials conducted at two different sites is consistently 0.3 or greater, they can be safely included in the same TPE. This method of grouping environments in the TPE should only be used if data from trials containing 20 or more varieties are available over several years.^7^

#### Be cautious in using this approach

First, it is necessary to make sure that the trials/locations are representative of the TPE (i.e., the farmers’ fields), and that crops are not grown only at the experiment station (often with water). Second, do not exclude trials that did poorly because of drought. Our experience from several analyses of METs shows that there is a large non-predictable component of GEI (associated with year-to-year variation), as well as a large error component. This makes it difficult to define consistent patterns for the grouping on the basis of locations (Cooper et al., 1999a) and requires large datasets to estimate frequencies of environmental types based largely on variable water conditions. Since our aim is to develop varieties with adaptation to these water conditions, we need to know more about the patterns of water supply and the types of drought. The GEI analysis needs to be supplemented with measurements of the water supply at the local level.

#### The process of defining the target population of environments is an ongoing one

Since most breeding programs conduct METs, a few modifications can improve the data for the continuing process of the TPE definition:

• Select “probe” varieties with contrasting differences in important traits (i.e., early or late, photosensitive or insensitive) as reference lines.
• Test these varieties under representative conditions, including farmers’ fields.
• Measure the water environment of the MET.

#### Monitoring water levels to characterize drought

Water supply can be monitored during crop growth to determine the timing and severity of drought to further define the TPE. The pattern of water level recorded over the season can be used to characterize three different types of drought:

• An early drought that occurs during vegetative growth.
• An intermittent mid-season drought that occurs between tillering and mid-grain filling.
• A late drought that occurs during flowering and grain filling.

In addition to knowing the frequency, it is also important to know the severity. For this, it is necessary to compare the yields under the drought and irrigated conditions or, if irrigation is not possible, choose a well-watered site such as the bottom of the toposequence.

#### Modeling the availability of water and the use of geographic information systems in the rain-fed lowlands to define the target population of environments

In the rain-fed lowland rice ecosystem, the TPEs are often defined by their position in the toposequence (hydrology). For example in Thailand, farmers’ estimates of yield reduction because of late-season drought were 45–50 and 15–20%, respectively, for the upper and middle levels on the toposequence.^8^ The national breeding program now uses the different positions on the toposequence to represent differences in the severity of drought in their testing program. A water balance model to predict available water has been developed and linked with geographic information systems (GIS) to characterize environments based on water availability (Inthavong et al., 2008).

#### How to determine the target population of environments for the upland rain-fed system

In upland rice, water availability for crop growth depends largely on rainfall patterns, rather than on total rainfall, and on land and soil properties that influence infiltration. The upland system is generally poorly buffered against variation in rainfall because it cannot store as much water as the lowland system. Short periods without rainfall (around 7 days) are most damaging if they occur just after sowing, when roots are poorly developed. Periods without rain can also cause spikelet sterility during the critical period from about 10 days before anthesis to 5 days after anthesis. As a general guideline for tropical areas:

• Flowering-stage stress will generally be significant after 7 days without significant (>5 mm) rainfall.
• For each additional day without rainfall during this critical stage, yield will decrease by about 10%.^9^ The water supply during crop growth can be estimated using a simple water balance model based on weather data and knowledge of soil texture and depth at a site. Starting from a soil at field capacity, use the following as a guide to make an estimate of water use:
• Water content at field capacity can range from about 10 mm (sandy soil) to 20 mm (heavy soil) per 100 mm of soil.
• Rice grows well until about 30% of the available water is extracted. This means that the crop will have 3–6 mm of water available per 100 mm of rooting depth.
• Rice roots of many *indica* varieties below 600 mm seem mostly ineffective in water uptake, so their maximum rooting depth is probably 600 mm.
• In soils with high acidity, plow pans, or other conditions that encourage surface rooting, rooting depth will be much less. Therefore the depth of effective rooting needs to be measured for the site.
If the roots extend to 600 mm, the crop can extract 18–36 mm of water, which is enough for 6–11 days of transpiration in the humid tropics during the vegetative and grain-filling stages, or 4–7 days of transpiration during the critical flowering stage. If the rooting depth is only 300 mm, a crop starting at field capacity can grow for only half this long before it begins to experience water stress.

It is recommended to use the rainfall and estimate of water use to develop a simple water balance for the crop to define the frequency and type of drought.

#### Choice and characterization of the testing environment

The TPE has to be used to define the breeding strategy; once the TPEs have been defined, a breeding strategy can then be developed for each TPE based on adaptation to the prevalent water supply and type of drought. In broad terms, that strategy includes selection for:

• Yield potential for favorable conditions.
• Drought escape (early maturing) for terminal stress.
• Drought tolerance for all stress conditions, but particularly intermittent stress.

However, when large year-to-year variation occurs in the type of drought, no one drought type can define the TPE. Under these conditions, breeders need to balance selection criteria to reflect the likelihood of each drought type in the TPE. The important point is to know which drought type occurred in each nursery and make sure that material that is well adapted to other frequently occurring drought types is retained among the selected lines. Otherwise, a cyclic pattern of genotypes adapted to different drought types can limit progress in selection.

Evaluation of the GEI helps to decide on the number of TPEs for the breeding program. In rain-fed environments, GEI or the tendency for genotypes to rank differently in different environments may be large. Under these circumstances, several TPEs, each served by different varieties, may be optimal. This is very different from irrigated rice, where the TPE can be very large, as in the example of IR36 grown on a large area. However, since each new TPE served will need additional breeding and testing resources, there will be a practical limit to the number of TPEs served by a breeding program. In some TPEs, the size of the target area will be inadequate to justify the resources required for a separate effort, and breeders must rely on the “spillover” of a variety from another TPE.

There is a trade-off between precisely defining the TPE and achieving enough replication within it. Thus, even when the TPE has been precisely defined, there will be random rank changes in variety means from site to site and from year-to-year, that cannot be explained by differences in water status. This is because many factors, such as pest damage, disease, and measurement error, routinely affect yield data collected in field trials. These “noise” factors are known to be very large in rain-fed lowland rice, and they can be overcome only through adequate replication within and across environments. If the TPE served by a breeding program is too narrowly defined, budget considerations will allow only one or a few trials to be conducted within each TPE. When genotype means are estimated from only one or two trials, least significant difference (LSD) values are very large, preventing accurate evaluations from being made and reducing progress from selection. In general, the TPE must be large enough to support three to five testing sites.

### Field experimental design^10^

The design should be precisely defined. A major departure from conventional (irrigated) rice breeding that is required in rain-fed systems is the need for early generation yield testing in selection environments (SE) that represent the TPEs and the large GEI within them. Replicate check lines must be used in early screening nurseries: in early generation screening trials, we are usually limited to very few environments. In fact, in some cases, the number of replications (*r*), locations (*l*), and years (*y*) may be only one. Even when all test lines cannot be replicated, one or more check lines should be replicated. Check lines in screening trials fall into two categories: probe lines that have well-known responses to specific stresses, and replicated checks that may be less well known but represent the test material as accurately as possible. Some guidelines for using replicated checks are:

• Lay out probe lines in a systematic way. The objective of these checks is to verify that the appropriate stress was in fact applied. For drought screening, a check line that is susceptible to the particular form of drought being tested should be used, and this might actually die under the applied stress.
• Identify plots for replicate check entries at regularly spaced positions in the field or screen layout. These positions must themselves be representative of the experimental space. In statistical terms, they represent a stratification of this space. They should not be selected to be at edges or along pathways, or in other non-representative areas. Border rows, plots, or pots as appropriate and necessary should in any case, protect them.
• The replicated checks should be allocated to the check plots according to a standard experimental design such as a randomized complete block (RCB) design or a Latin square design (see CROPSTAT tutorial^11^ on “Randomization and layout of experimental designs”). The resulting nursery is then described as being laid out in an “augmented RCB” or “augmented Latin square” design as described below. If the field contains a single identifiable gradient, then an RCB with blocks perpendicular to the gradient is appropriate. For spatial control in two directions, the Latin square is better.

The main objective of the replicated checks is to quantify spatial variability in the test environment and adjust the measurements of the test lines accordingly. A desirable byproduct of using replicated checks is an estimate of measurement error and, indeed, if the checks themselves are interesting test material, extra valuable information is obtained on those particular lines [see CROPSTAT tutorial (see text footnote 12) on “Single-site analysis for variety trials”]. We recommend the use of augmented designs that have been developed to overcome the serious drawbacks of unreplicated trials, such as a lack of control of field variability, and no estimate of error for comparing entries (Federer and Raghavarao, 1975).

In advanced yield trials and METs, the main objective is to increase the number of environments where lines are evaluated. With limited resources it is preferable to increase the number of sites rather than the number of replications in any one trial. To do this, we use designs that are more efficient than the RCB designs such as modern alpha lattice designs. However they require specialist computer programs for their design and analysis. Some guidelines for effective METs are:

• Increase the locations rather than the replications to maximize the chance of testing under drought conditions.
• Choose locations that are likely to experience the relevant drought stress.
• Use a lattice design with only two replications and small blocks (<less than 10 plots per block) at each location (see CROPSTAT tutorial^8^ on “Randomization and layout of experimental designs” and on “Single-site analysis of variety trials” for examples of how to use classical simple lattice designs).
• Use data from drought trials even if coefficients of variation (CVs) are high (provided that the trials were well-conducted).
• Do not use yield data from locations that do not experience the target drought stress for the TPE, unless the wish is to use them as an estimate of yield potential.^12^

### Breeding to improve yields under drought: from the SE to the farmer’s fields and how to increase response to direct selection for yields

The SE must be representative of the TPE. Performance in the TPE and the SE can be thought of as correlated traits expressed by a single genotype in separate environments. This relationship is measured as the genetic correlation (*r*_G_). Thus, the *r*_G_ is an indicator of the accuracy with which performance in the TPE can be predicted in the SE. An *r*_G_ value of 0 indicates that there is no association between performance in the selection and target environments. An *r*_G_ value of 1 indicates that the SE is perfectly predictive of performance in the TPE. Therefore, before embarking on a controlled-drought screening program, the breeder needs to test the assumption that the performance in the controlled experiments is predictive of performance in the research station field (*r*_G1_) and that performance in the research station field is predictive of performance on-farm under farmer management (*r*_G2_). To maximize *r*_G_ between the SE and the TPE:

• Ensure that conditions at the research station (nursery and trials) are similar to those in farmers’ fields. Note that selection is often conducted at research stations under management regimes that are not representative of those used by farmers. This type of selection may be justified in terms of selecting for yield potential or maximizing the precision of yield trials, but breeders must ensure that performance on-station is predictive of performance on the farm.
• Use two kinds of screening trial, one that predicts performance in drought years and one that predicts performance in favorable years. For the design of the managed-drought screening trial see the section “Water-stress management and characterization” below. Note that nurseries in which managed levels of stress are purposefully applied are useful in ensuring that *r*_G_ is maximized for stresses, such as drought, that occur sporadically in the TPE. It is important to verify that the results of managed-stress trials really are predictive of performance on-farm.
• Select directly in the target environment, that is, on-farm. For on-farm screening, the correlation between performance in the selection and target environment is necessarily 1, assuming that representative farmer-cooperators have been chosen. Therefore, on-farm screening should be a component of all breeding programs in which any uncertainty exists about the predictive power of on-station screening. Note that on-farm trials can be expensive and imprecise because of variability caused by weeds and low fertility, and are subject to a high risk of failure. Consequently, on-farm testing programs must be carefully designed and conducted to avoid wasting money and time, and to maximize the reliability of the data obtained. Use the robust experimental designs discussed earlier.
• Irrigate only if the objective is to measure yield potential.
• Use data from trials affected by drought even when the coefficient of variation (CV) is large; the inherent variability of stressful environments is often high (Atlin and Frey, 1989). This has important implications for the use of data from METs and on-farm trials in selecting drought-tolerant materials. Often, trials with high CVs are omitted from the analysis. However, these are frequently the trials in which stress was most severe. Omitting high-CV trials almost always introduces bias into the sampling of the TPE toward more favorable environments. This bias can be avoided by not using an arbitrary CV value as a criterion for accepting or rejecting a certain on-farm or off-station trial. If no obvious errors have been made in layout or data collection, results from low-yield, high-CV trials should be retained. These are often precisely the trials that are the most informative about cultivar performance in stressful environments.
• Select genotypes that perform well under both drought and well-watered conditions. Varieties that perform well in both types of SE can generally be identified because *r*_G_ across drought stress levels is usually positive in other crops (Atlin and Frey, 1989; Bänziger et al., 1997) and there is evidence that *r*_G_ is also usually positive (sometimes with a low value) in rice grown under a range of water-stress environments (Lafitte and Courtois, 2002; Pantuwan, personal communication). Selection intensity must be high. Drought-tolerance breeding programs must be large to make progress. In most rain-fed rice breeding programs, only a few lines (usually fewer than 50) are tested in the replicated MET at several locations, although this is the selection phase most responsible for making gains in stress environments. If little selection pressure for yield under drought stress is applied, little progress will be made. For a small rain-fed rice breeding program focusing on drought tolerance and producing 1,000 new F_6_ or F_7_ lines per year from its pedigree breeding program, an appropriate distribution of effort might look something like the scheme below:
• preliminary managed-stress screening: *N* = 1,000
• preliminary replicated yield testing under stress: *N* = 200
• METs – advanced lines: *N* = 100
• participatory on-farm testing: *N* = 20.

The following techniques can increase the number of plots and, therefore, the number of entries using the same resources:

• Use augmented experimental designs that maximize the number of entries for given resources.
• Use micro-plots and visual rating scales judiciously (see later section).
• Use screening methods that are inexpensive and able to handle large numbers.

Broad-sense heritability (*H*) must be maximized through careful management of drought screening nurseries and by high levels of replication within trials and across sites and years. There are several ways to increase *H*:

• Increase the number of replicates per trial.
• Increase the number of trial locations.
• Increase the number of years of testing.

It is important to reduce the error $\left(\sigma_{\text{E}}^{2}\right)$ variance to detect real differences between lines. In our experience, the genotype-by-location-by-year $\left(\sigma_{\text{GLY}}^{2}\right)$ and the error $\left(\sigma_{\text{E}}^{2}\right)$ variance are the largest contributors to random noise in field trials. The contribution of $\left(\sigma_{\text{E}}^{2}\right)$ can be reduced by choosing uniform test sites, increasing within-site replication, adopting improved methods of controlling within-block error (for example, lattice designs or neighbor analysis), or increasing the number of locations or years of testing. The contribution of $\left(\sigma_{\text{GLY}}^{2}\right)$ can only be reduced by increasing the number of tests across locations or years. This is expensive and must involve:

• cooperation among research centers in collaborative networks for the early stages of yield testing, rather than extensive testing at a single center until advanced stages (Cooper et al., 1999b)
• increasing the number of test locations rather than the number of replications at each site.^12^

Increasing the number of replicates (without increasing the number of trials) is less expensive but also less effective in increasing heritability!

### Water-stress management and characterization^14^

One of the major limitations to the improvement of rice for drought-prone areas has been the lack of appropriate methods to impose drought routinely and reliably in order to select more tolerant lines. Many methods have been used to impose drought in order to have a better understanding of the mechanisms that lead to higher yields and the traits that can be selected for drought tolerance. However, few have been evaluated to assess their predictability of performance in farmer’s fields (see more on this later). Each has a strength and weakness as outline in Table 5. Therefore, care is needed in deciding which approach to use. We advocate more studies to validate that the testing environment predicts performance in farmer’s fields.

**Table 5:** **Evaluation of different field devices for genotype study/screening in response to drought**.

Field devices for drought study	Cost	Strengths	Limitations	Suitable climate and soils	Reference
Late planting with drainage in rainy season trial	Large uniform field management	High chance of reproductive and terminal drought	Photoperiod non-sensitive	Semi-arid tropics	Pantuwan et al. (2002a)
Dry-season trial	Large uniform field management	High chance of drought, vegetative drought	Photoperiod non-sensitive, genotype-by-season interaction	Semi-arid tropics	Pantuwan et al. (2004)
Line-source sprinkler	Equipment, water source, monitoring	Different water regimes	Wind, space	Semi-arid to arid climate	Garrity and O’Toole (1994)
Rainout shelter	Construction	All types of drought	Space, cost		Lilley and Fukai (1994)
Greenhouse	Construction	All types of drought	Space, cost, rhizosphere differences (small and loose)		Yadav et al. (1997), Wade et al. (2000)
Root restriction	Rhizosphere manipulation	Evaluation of non-root traits^a^	Space	Hardpan, simulated lowland	Kato et al. (2007)
Raised bed	Rhizosphere manipulation	Dry surface soil (interrupt capillary water)	Space	Sub-humid climate	Kato et al. (2007)

*(Source: Kamoshita et al., 2008)*.

*^a^Restriction of the root zone removes the advantage of deep rooted varieties that would be expressed if no restriction; in most puddled lowland fields roots are restricted*.

#### Start with a uniform field and apply all inputs uniformly

When fields are well irrigated, they often appear uniform. However, as drought develops, differences in topography, slope, soil texture and field history can have a large effect on plant growth. Choose a level field with minimum variation in soil depth or texture. Not all the variation in a field can be seen from the surface; observations of weed or crop growth in a previous season can give hints of problems. A transect of soil cores or soil impedance readings can also indicate below-ground variation. If irrigation is applied, it must be uniform in depth. Replicates or incomplete blocks should be placed inside a basin. If sprinklers are used, irrigation must be applied when there is little wind. All sprinkler heads must throw the same amount of water, so the pump pressure must be high enough to pressurize the system evenly. Sprinkler heads must be cleaned and checked, and leaks should not occur within plots. Other management practices such as the application of fertilizer and weed control should also be carried out uniformly. If it is found that uneven drying still occurs in the field, a visual score of soil drying can be given to each plot when differences are obvious, and this score can be used to adjust for field differences. Statistical designs are available that can also help deal with variability, but there is no substitute for starting with a good, uniform field.

#### Know what happened

Whether managing irrigation or relying on natural drought periods for stress, the essential measurements needed to characterize the environment are depth of standing water (in lowland fields), depth of the water table, and daily rainfall:

• The simplest measure is to record the presence or absence of standing water weekly. A late-season drought can be identified by the last date of the standing water relative to the flowering date of the variety.
• A measure of the depth of the water above and below the ground is more informative. For an accurate measure of the above ground water, use a “slant meter”; for below the ground, use a PVC tube.
• Use a minimum of three recording stations for each trial located across any perceived water gradient.
• Make some additional measurements. It is useful to know pan evaporation and this can be measured from a central station in a region. For upland experiments, it is useful to know soil moisture tension, which can be measured inexpensively using a tensiometer. For guidelines on making groundwater wells and tensiometers, see Mackill et al. (1996).
• Remember that many potentially useful datasets cannot be interpreted because no one knows whether drought affected the experiment or not. Observations of leaf rolling in check cultivars can provide good evidence of when water stress began. It is critical to know both the dates of disappearance of standing water in lowland fields and the amount of water in upland experiments. If the water table is at a depth of 1–1.5 m, it can provide an additional source of water to the crop; so check for groundwater depth.

#### Keep out unwanted water

To apply stress consistently, there must be a way to limit water input to the plots. This can be done by the following means:

• Sow at a time of year when a good chance of low rainfall is expected (provided that this season is representative of the regular season in the target environment).
• Use a rain exclusion shelter. Such shelters are expensive to build and maintain, so these are usually used only for small experiments. The temperature under shelters tends to be higher than the outside air temperature. This may affect crop flowering date and can, in some cases, result in high-temperature damage. Monitoring of air temperature will allow interpretation of the results.

Check for water from underground sources, especially if there is lowland rice nearby. To avoid entry of water from adjacent wet areas, between the experimental field and the source of free water, it is necessary to dig a ditch that is at least 40 cm deeper than the expected root zone. This ditch will intercept water moving into the field, and the water must then be drained away. At upland sites, lateral water movement is not usually more than about 1 m but, depending on the irrigation method, it may be necessary to have wider borders.

#### Remove water at the desired time

In rain-fed lowland experiments, the soil is generally saturated before stress begins, and the field is then drained to allow the development of drought. The number of days it takes for drought to develop depends on the moisture-holding characteristics of the soil, losses from seepage and percolation, and the amount of water transpired by the crop. Thus it is necessary to conduct an initial experiment to see when to remove water to induce stress at the desired time. Remove water at a developmental stage of a check variety. With experience, it is possible to estimate the number of days this will require in the experimental field. For a fully developed crop growing in a heavy clay soil at IRRI, it takes about 10 days for a field to dry from saturation to near field capacity. After about 1 week more, some leaf rolling can be observed. This means that it takes about 20 days for stress to develop after the field is drained, and would take more time if the crop were small. In contrast, sandy soils dry much more quickly and stress can develop within 14 days or so.

In upland experiments, it will take much less time for stress to develop after rainfall or irrigation stops. If root depth is shallow (25–30 cm), the amount of water available to the crop between field capacity (about 10 kPa) and 20 kPa is only adequate for a few days of transpiration, and irrigation must be applied every 2–3 days in control plots. Stress will begin almost immediately on the withholding of the irrigation.

It is also possible to apply a mild continuous stress by simply reducing irrigation frequency. This has the advantage that it has a similar effect on genotypes with different flowering dates, and the stress treatment is not affected much by minor rainfall events. However, a mild continuous stress is not very effective in separating lines for some traits that require more severe stress, such as flowering delay and leaf drying.

#### How severe a drought stress?

Aim to reduce yield by almost 50%. One reason for this is that *r*_G_ for line means estimated in trials with only slightly different stress levels is likely to be very close to 1.0. Another reason is that severe stress, when skillfully and uniformly applied, can amplify genetic differences between lines. For example, if uniform and severe drought stress can be applied to rice breeding lines at flowering, some highly susceptible lines simply do not flower. This is a large, visible genetic response that can make it easy to eliminate susceptible genotypes.

#### Conduct a companion nursery under well-watered conditions

In addition to the controlled-drought SE, it is very useful to have a companion nursery with well-watered conditions to estimate the yield potential of the genotypes:

• Estimate the severity of the controlled environment as the mean reduction in yield between the well-watered and the drought nursery.
• Avoid water deficit in the uplands; irrigation is usually applied when the soil moisture tension at 15 cm depth reaches about 20 kPa.
• Maintain free-standing water in the well-watered rain-fed lowlands.

#### Correct for differences in flowering date

Rice is especially sensitive to stress around flowering. This means that a line that flowers shortly after the field has been drained will be much less affected by stress than a line that flowers later. One option is to place genotypes in early, middle, and late maturity groups, and stagger the planting dates so that all genotypes flower at the same time. This requires good information on flowering time and is difficult to manage. Another possibility is to stratify the entries based on the flowering dates of the well-watered plots, and select lines that are less affected by stress within each group. If there is a clear linear relationship between stress yield and flowering date, a drought response index (DRI) can be used (Bidinger et al., 1987).

This means regressing stress yield on flowering date in the control, and finding the predicted yield as follows:

Predicted yield = *a* + *b* (flowering date) And the DRI is calculated as: (observed yield-predicted yield)/standard error of predicted yield.

### Other points to consider

Dry-season screening is, in most parts of the world, equivalent to out-of-season screening. Fields that are sown out of season are generally much more susceptible to insect, bird and rodent attack because other food sources are unavailable. There are also climatic factors to consider, such as low temperature, high radiation and low humidity. Because of these factors, performance in a dry-season nursery may not accurately predict yield potential for a variety targeted to the wet season. The main purpose of the dry-season nursery is to obtain additional information about drought tolerance. This information can be combined with other data from wet-season screening in a selection strategy.

When rice is grown repeatedly in upland fields, yield potential often declines markedly after the first crop or two, perhaps because of nematode accumulation, micronutrient deficiencies, or other unknown factors. If a field is developed as a long-term screening site, it should be large enough to allow part of the field to be rotated with a non-rice crop each year.

### Phenotyping (traits)

#### Which secondary traits are useful?

There must be a relationship between the secondary trait and grain yield in the target environment. The traits expected to be of value in some drought-tolerance breeding programs are shown in Table 6. However, even when this relationship is found, that is not enough to show that breeders should use the secondary trait. For breeders to use the trait, the expected progress from selection using the secondary trait and yield together must be greater than the progress made using grain yield alone. Kamoshita et al. (2008) provide a review of the broad-sense heritability of the main traits proposed for use in selection for drought tolerance in rice. Based on an earlier assessment by Lafitte et al. (2003) the recommended traits are:

• *Flowering/maturity date* (useful for predictable terminal drought): Rice is extraordinarily sensitive to water deficit from about 12 days before 50% flowering to about 7 days after flowering. If the pattern of water deficit is predictable in a given region, selection for a flowering date that does not coincide with the period of water deficit is a very effective way to improve drought tolerance. The limitations to this approach are that very early varieties may suffer a yield penalty in good seasons, and that this approach works only where the timing of the water stress is quite predictable. As well as avoiding drought at critical growth stages, there may be an additional advantage to comparative earliness. Early materials sometimes tend to have a more stable HI than later ones.
• *Flowering delay* (useful for intermittent mid-season drought): When rice experiences a water deficit before flowering, a delay usually occurs in flowering date. Lines with a longer delay will tend to produce less grain, even if the water stress is relieved later. The length of the delay is partly related to the amount of stress the line experienced, but there is also genetic variation in how much delay results from a given level of stress. The reason for the delay in flowering is not fully understood.
• *Percentage of fertile spikelets*: When stress occurs near flowering, i.e., the most sensitive growth stage, the main yield component affected is the percentage of fertile spikelets. The genetic correlation between yield under stress and this trait is very high, and the heritability of spikelet fertility is less affected by stress than is the heritability of grain yield. The way that spikelet fertility is affected by drought at flowering is quite specific, so it gives clearer information on genotypic response to stress than does yield, which is the integrated result of many processes that occurred over the season. However, many factors other than drought can affect spikelet sterility, and some of these, such as stem borer damage, interact with drought. Experiments should be monitored for possible confounding factors.
• *Leaf-death (desiccation or “firing”) score*: Leaf water deficit can be reduced further beyond the point of turgor loss, reaching the point of tissue death. Leaf tissues may die (showing desiccation) because of extreme loss of water or because of heat stress when the leaf temperature rises as a result of inadequate transpirational cooling. Unlike leaf rolling, leaf desiccation is irreversible. All leaves in the canopy should be observed when leaf death is scored. Desiccation may not occur throughout a given leaf in a uniform fashion, unless the water deficit is acute. More typically, it begins at the tip of the leaf, which is usually under greater water deficit than the basal part closer to the stem. If the timing and severity of drought in the screening environment are similar to those of the target environment, leaf drying can be correlated well with yield under stress.

**Table 6:** **An assessment of selected secondary traits expected to be of value in some drought-tolerance breeding programs**.

Trait	Relationship to stress yield	Growth stage for selection	Earliest generation for selection	Technical difficulty of selection	Heritability
Flowering/maturity date (Babu et al., 2003)	Depends on reliability of stress timing; effective for predictable and terminal stress	Flowering	Single plants at F_2_	Easy	High heritability (ca. 0.9)
Flowering delay (Kumar et al., 2007)	High for stress at flowering	Flowering	When available, seed is sufficient for a small plot	Easy if water can be controlled to provide uniform stress	Moderate heritability (ca. 0.6)
Percent fertile spikelets (Babu et al., 2003; Kumar et al., 2007)	High for stress at flowering	At or near maturity	Single plants at F_2_	Labor-intensive; error-prone; requires control of water	Moderate heritability (ca. 0.6)
Leaf-rolling score (Babu et al., 2003)	Negative and moderate	Vegetative	Single plants at F_2_	Easy if water can be controlled to provide uniform stress	High heritability (ca. 0.8)
Leaf-death score (Yue et al., 2005)	Negative and moderate	All stages	Single plants at F_2_	Easy if water can be controlled to provide uniform stress	Moderate heritability (ca. 0.7)
Canopy temperature (Yue et al., 2005)	Negative and fairly high if maximum stress occurs near flowering	Pre-flowering during full ground cover	When available, seed is sufficient for a small plot	Medium	Fairly low heritability (ca. 0.2) unless climate is very stable and vapor pressure deficit (VPD) is large

*Source of assessment: Lafitte et al., 2003; more detail assessments of the heritability for each trait is found in the reference for each trait. Practical instructions on the measurement of these traits are provided in Measurement of Secondary Traits: Some Practical Considerations*.

### Conclusion

#### Choice of parental material

Atlin (2003) notes that choosing parents is one of the most important steps in a breeding program. No selection method can extract good cultivars if the parents used in the program are not suitable. Although breeders have different approaches to parent choice and have achieved success in different ways, many successful crosses have some common features that can be recommended:

• Use at least one locally adapted, popular cultivar as a parent. This helps ensure the recovery of a high proportion of progenies with adaptation and quality that are acceptable to farmers. If quality requirements are very important and if the local variety is highly preferred by farmers, a backcross to the local variety may be required to reach an acceptable level of quality.
• Choose each parent to complement the weaknesses of the other. For example, if both parents are susceptible to an important disease, it is highly unlikely that many offspring will be resistant. Thus, when breeding for drought tolerance, avoid parents that are highly drought-susceptible.
• Use improved modern varieties in crosses with an adapted parent. Often, elite modern varieties have high yield potential and many disease-, insect-, and abiotic stress-tolerance genes that local ones lack.
• If no drought-tolerant cultivars are known, evaluate a diverse range of cultivars and advanced lines for the characters identified for the TPE, including the specific characters for drought tolerance. This will mean testing the potential parental material under controlled drought.

Researchers in Thailand, Cambodia, and Laos have screened local materials for drought tolerance; they used DRI to normalize the effects of yield potential and flowering date on yield under drought stress. DRI ranges from −2 to +2, and values greater than 1.4 may be considered as drought tolerance. When several experiments are considered, the mean DRI of the drought-tolerant genotype may be below 1.4, with the actual value depending on the consistency of performance across the experiments. The DRI provides a better estimate of the contribution of drought-tolerance traits to yield under drought, independent of those for yield potential and flowering. However, this estimate is prone to high errors and should be considered mainly as supporting evidence. These researchers screened a total of 1,279 rice genotypes including a large number of landraces for drought resistance in 34 experiments across the three countries. Drought was imposed (i.e., controlled drought) in 76% of the trials. The project validated the use of DRI for grain yield and spikelet fertility as important drought traits. DRI heritability ranged from 0.39 to 0.88, and from 0.31 to 0.77 for grain yield and spikelet fertility, respectively. In each country, the selected donor lines were crossed to local recipient cultivars with a high yield potential and/or good grain quality attributes. A total of 85 populations (40 for Thailand, 19 for Laos, and 26 for Cambodia) were developed that were derived from single-seed descent (SSD). In Thailand, a number of populations were backcrossed to the recipient parent to form NILs. Five RIL populations in Laos, eight in Cambodia, and six in Thailand were selected based on the performance of the putative drought lines, and are being carried forward. These, plus some of the original populations, are now part of the routine breeding program of the three countries. The progenies (F_6_) will be phenotyped for drought response, and superior lines will enter the routine advanced testing trials (Report to Rockefeller Foundation, 2006).

#### Early generation yield testing in the target population of environments

A major departure from conventional (irrigated) rice breeding that is required in rain-fed systems is the need for early generation yield testing in SEs that represent the TPE (Atlin, 2003; Jongdee, 2003). The aim of the breeding program is then to develop fixed lines for early yield testing at a large number of sites (direct selection for yield) and under controlled-drought conditions (indirect selection). A number of strategies can be followed:

• Fix lines through SSD. The main goal is to fix the lines with minimum selection. Where facilities are available to control day length (and when using photoperiod-sensitive materials), up to three generations per year can be produced using rapid generation advance (RGA), thus reducing the time to develop fixed lines (F_5_ and later) for yield testing.
• Fix lines through the normal process of single plant selection within the F_2_ and later generations in the bulk method. Usually, two generations are developed each year by the use of an off-season nursery. This provides an opportunity to select for characters that are more highly heritable – selection is based on a single plant or progeny row and one observation. It also creates a danger that selection, particularly under irrigation or in the off-season nursery, will not be representative of the TPE.
• Select for traits such as maturity and height (main season) and disease resistance only in the early generations, if the desirable agronomic traits have been identified with farmers’ priorities in mind. For example, breeders may select short materials because of their high yield potential, but farmers may not accept these because of various problems such as poor weed competition and low straw yield.
• Select under drought conditions in the early stages. Many plants in a segregating population may not produce any seed because of susceptibility to drought. Since the heritability of drought tolerance is usually low, it will be beneficial to practise this type of selection for more than one generation. Many breeders find that the bulk method of breeding is suitable for this type of environment, and requires fewer resources than the pedigree method.
• When fixed lines are developed (F_5_ or later), seed supplies are sufficient for replicated testing. This will allow more flexibility in conducting METs in the TPE.

#### All breeding programs should include participatory on-farm trials

To ensure that selection has been effective and that progress made at the station will be transferable to the farm, on-farm trials managed by farmers should be part of the testing of a new cultivar (Atlin, 2003). In such trials:

• Include as many cultivars as possible in participatory testing by farmers in their fields.
• Consider the use of “mother-baby” trials (Bänziger et al., 1997) to maximize the number of genotypes tested.
• Run participatory trials concurrently with advanced METs.
• Test for grain quality, in consultation with farmers from the TPE. This is cheaper than replicated yield testing. Hence, quality screening should be done before METs to discard varieties with quality unacceptable to farmers.

## Conflict of Interest Statement

The authors declare that the research was conducted in the absence of any commercial or financial relationships that could be construed as a potential conflict of interest.

## Footnotes

^1^In the same study Pandey et al. (2007) showed that for northeast Thailand and southern China, the losses were smaller, averaging less than US$20 million per year (or less than 1.5% of the value of output).

^2^The term “drought-tolerant variety” as used here refers to a variety that produces a high grain yield relative to other cultivars under drought stress. This definition is as given by Atlin (2003).

^3^See: www.iris.irri.org

^4^http://www.oryzasnp.org/

^5^Reprinted from Lafitte et al. (2003).

^6^Reprinted from Fischer et al. (2003b).

^7^Rajatasereekul et al. (1997) used this approach to define three broad domains for the rainfed systems of Thailand and Lao PDR and, from that, the duration of preferred varieties.

^8^Boonrat Jongdee (see The Thailand Experience of Integration of Direct Selection for Grain Yield and Physiological Traits to Confer Drought Resistance).

^9^Courtois and Lafitte (1999) have used this approach for a regional characterization of the uplands.

^10^Reprinted from McLaren (2003).

^11^http://www.riceworld.org/science/software/cropstat.asp

^12^Reprinted from Atlin (2003).

^14^Reprinted from Lafitte (2003).

## Appendix

### The thailand experience of integration of direct selection for grain yield and physiological traits to confer drought resistance

The rain-fed lowland is a major rice ecosystem in Thailand with an area of approximately 5.7 million ha, more than 60% of the total rice land of Thailand. Rainfall is bimodal and drought may develop early and late in the growing season. The early season drought occurs in most areas, affecting the time of transplanting of seedlings and the growth of direct-seeded rice. Late-season drought develops at the end of the monsoon season in most years in the northeast, particularly on upper part of the toposequence of the paddy, where there is more water loss from soil percolation and lateral water movement. Early season drought is more frequent than late-season drought, but yield loss is more severe in the latter. Thus, the target of our breeding program is the development of late-season drought-tolerant cultivars. The following describes our approach to screening and breeding for drought tolerance. To date nine breeding lines of traditional and improved germplasm have been selected and used as donors for drought tolerance. Single and backcross populations had been developed. Presently, 10 lines which have been selected under field screening are being tested under farmer fields in target drought-prone areas.

#### The breeding approach

The breeding approach has been changed to increase efficiency and shorten the selection process. The change is based on recommendations from the Australian Center for International Agricultural Research (ACIAR) project on “Plant breeding strategies for rain-fed lowland rice in northeast Thailand and Laos” (Cooper et al., 1999a,b). The previous breeding program took 12–15 years, now the cycle is completed in 10–11 years. There are three major phases of the selection cycle: intra-station (local, on-station selection); inter-station (across 13 stations, on-station selection); and on-farm selection. In the previous breeding system, selection was carried out mainly in the intra-station phase and most lines were discarded based on visual selection and on the results from yield testing at a single location (i.e., local adaptation). Only a small number of lines relative to the total generated from the crossing program were selected for subsequent inter-station (wide adaptation) and on-farm performance. This selection system made it difficult to identify high yielding lines at the farm level due to a large GEI for grain yield (Cooper et al., 1999a). One of the recommendations for the breeding system was to replace the intra-station phase with early generation inter-station yield testing of F_4_ bulks in order to select for wide adaptation at an earlier stage of the selection process. However, the F_4_s are still segregating for flowering date and this causes some error in estimating grain yield. We modified the recommendation to develop our new breeding system which tests large numbers of F_7_/F_8_ in the inter-station (multilocation) trials. We use the RGA technique at the intra-station phase to save time. Recently we have again modified the selection to incorporate on-farm testing earlier in the selection process. The details of these changes in the selection process are described by Jongdee (2003).

#### Definition of the target domain

The majority of the lowlands are in the northeast and north, and are classified as shallow-favorable and shallow drought-prone. We used GEI and cluster analysis of grain yield from multilocation trials to further define our TPE. However, groups of environments changed from year-to-year, resulting in a large genotype-by-year component of the GEI, and it was difficult to define genotype-by-location groupings. Recently, we changed the system of defining the TPE based on our work with farmers. We conducted a farmer participatory workshop for production improvement for rain-fed lowland rice in north and northeast Thailand and, from this, identified the target domains based on hydrology of rice paddies. Three levels of the paddy toposequence are identified – upper, middle, and lower terrace paddies – and these three water environments are included in the test locations in each region. Drought may occur at any time during the growing season as shown in Table A1, but our focus is on improvement for the intermittent and late-season drought. The upper terrace paddy can be defined as unfavorable conditions, in which drought can develop at any growth stage. The middle terrace paddy can be drought prone, where rainfall is variable and soils are light in texture. In other areas, the middle part of the toposequence can be considered as favorable. The lower terrace paddy can be defined as less favorable, because drought may develop in the early season followed by a sudden flood. The estimates by farmers of yield reduction due to late-season drought were 45–50 and 15–20% for the upper and middle terrace respectively. We use the different positions of the toposequence to provide differences in the severity of drought in our testing program.

**Table A1:** **Use of the position on the toposequence to define the types of drought occurrence and the target population of environments (TPE) for the breeding program**.

Position on the toposequence	Type of drought occurrence	Yield loss in the TPE
Upper	Early, intermittent and late drought	Late drought causes 45–50% yield loss
Middle (drought-prone)	Early and late drought	Late drought causes 15–20% yield loss
Middle (favorable)	Early drought	Minimal yield loss
Lower	Early drought and sudden flood	Minimal yield loss in drought; higher risk of loss from flooding

#### The selection strategy

The different selection criteria used for developing cultivars for each of the TPE defined by the upper, middle, and lower terraces are shown in Table A2. Phenology, particularly flowering time, is the most important trait for avoiding the late-season drought in each of the different domains. Flowering must occur before the standing water in the paddy disappears. Thus we select three flowering groups for the different domains of the toposequence:

• early maturing: flowering around mid-September to beginning of October
• intermediate maturing: flowering around mid-October
• late maturing: flowering around late October.

We select directly for yield in the multi-site selection program (described below) and we manipulate the water environment at a few sites in order to measure the drought-tolerant traits of flowering delay, spikelet sterility and, increasingly, for leaf water potential (LWP).

**Table A2:** **Selection criteria to develop varieties for each target domain**.

Target domain	Cultivar requirement	Drought traits	Selection strategy
Upper	Early maturing drought tolerance Low number of tillers	Maintenance of LWP Less delay in flowering Low spikelet sterility	Select for yield under the test location
Middle (drought-prone)	Intermediate maturing photoperiod sensitivity drought tolerance Intermediate height	Maintenance of LWP Less delay in flowering Low spikelet sterility	Select for yield under the test location
Middle (favorable)	High grain yield Intermediate height	Nil	Select for potential grain yield
Lower	Late maturing photoperiod sensitivity submergence tolerance	Nil	Select for yield under the test location

#### Water management to simulate late-season drought (at three of the test locations)

The drought screening trials under water-managed conditions are conducted in the wet season, in which the seeding is delayed by 2–3 weeks compared to the normal planting time. This increases the chance of the development of a late-season drought. Also, the standing water is drained from the field 2 weeks prior to flowering time, to further induce drought stress during the targeted growth stage. The water is drained from the field when the earliest lines have reached the flag leaf stage. If necessary, irrigation water is added to ensure free-standing water prior to the flag leaf extrusion. Measurements in this trial include grain yield, spikelet sterility, and flowering date. The main measure of drought resistance used to complement direct selection for yield is spikelet sterility. We measure the percentage of sterile spikelets from panicles that are randomly harvested in each line, and which are grown in the controlled-stress trial. Variation in flowering date among the test lines causes differences in the severity of the drought stress and, thus, the spikelet fertility. To adjust for this effect, we compare spikelet sterility and grain yield among lines within the same maturity group. Because drought can occur at any time during the growing season, we record the pattern of water supply and the severity of the drought. We measure the standing water in the paddy as an indicator for drought development, and the level of underground water below the soil surface as an indicator for the severity of drought. We use a slant meter to measure the surface water and a piezometer to measure the water underground. All observations are made on a weekly basis.

#### Crossing and rapid development of fixed lines for yield testing

Only a few research stations are involved in the development of lines for yield testing. Photoperiod-insensitive materials are advanced for one or two generations in the same year by growing them in the dry season. In photosensitive materials, we use a dark room to induce flowering as part of the RGA methods.

In order to reduce the number of materials before RGA, selection can be conducted on the F_2_ generation for characters with high heritability such as height, plant type, flowering time, and grain size. (Note that there is no selection while under RGA).

#### Direct selection for yield and for drought-resistant traits at the station

Thirteen research stations across the north (five stations) and northeast (eight stations) are involved in the multilocation yield-testing program. The trials are conducted under two conditions of water availability: the water regime of the normal rain-fed lowlands in 10 stations, and a water regime that is manipulated to simulate late-season drought in three stations (two in the northeast and one in the north). The objective of this selection is to evaluate families for grain yield under normal rain-fed and late-season drought conditions.

The F_7_ lines developed from the intra-station selection (i.e., mainly for plant and grain type), are evaluated in two steps: an inter-station observation trial and an inter-station yield trial. The inter-station observation trial contains a large number of lines (200–300) grown in two replications and in plots of four rows, 2 m in length. In some cases, the lines are grouped on flowering time and form a separate trial, with each trial containing a set of check varieties that have been selected for their known response to different water environments. An alpha-plus experimental design is employed. The data are analyzed using REML, SAS, and GenStat. The selection in the inter-station observation trials is based on grain yield under normal and manipulated late-season drought. The first analysis is of grain yield data from the normal water regimes from each of the 10 stations. The data are analyzed by site and also in a combined analysis across the stations. The lines are grouped based on the GEI analysis for yield into different patterns by cluster analysis. The group(s) of lines that perform well at most environmental sites are selected, and the group(s) that have low grain yield in most environmental groups are discarded.

Because there is variation in flowering time among test lines and thus the timing of drought influences the yield, the second analysis is conducted for lines within the selected groups. Individual lines are selected based on spikelet sterility percentage and on grain yield under the manipulated late-season drought, bearing in mind the variation in the flowering date. Lines with resistance to the major diseases and insects pest, and with appropriate grain chemical quality are selected at this step as well.

The inter-station yield trial is conducted across the same stations in the north and northeast, using the same experimental design as that of the inter-observation trial, but with three replications. The plot size is expanded to five rows, 5 m long. The lines may be grouped by flowering date if there are a large number of lines in each flowering group. The grouping facilitates trial management of the timing of fertilizer application and of bird control, and it allows for the adjustment of the effects of different flowering times (and therefore different levels of stress) on grain yield. The selection of lines is based on the grain yield under rain-fed conditions, and also under the manipulated late-season drought. The approach is the same as described for the observational trials. Again, there is selection for resistance to important insects and diseases, and for chemical grain-quality characters.

#### Selection at the farm level

Our previous on-farm trials included only four to six lines with different flowering times, and favored the selection of lines for shallow-favorable conditions which are not representative of farmer fields. More recently, Inthapanya et al. (2000) have suggested more rigorous testing in farmer fields representative of their risk of drought and of the levels of fertility. We now conduct two stages of on-farm trials, the first with a large number of lines in each of the three flowering groups of our target domain, in which 20 lines are grown with a small plot size (6–8 rows per plot). The second is conducted with a small number of lines with a large plot size (16 rows per plot). The farmers’ evaluation of agronomic characters (panicle size, grain color, etc.), is conducted during grain filling. The selected lines are tested for eating quality at harvest using 15–20 farmers at each site.

#### Selection of parents

Now that we have modified our routine breeding program we are focusing on the selection of parental material based on more in-depth screening of sound physiological traits. We are selecting drought resistance donors based on the following criteria:

• maintenance of LWP
• drought score
• DRI
• delay in flowering
• spikelet sterility.

The trials used to phenotype the progenitors are conducted in three locations, two in the northeast and one in the north. We use two screening systems to induce drought: a line-source sprinkler and the water drainage technique applied before flowering, as described earlier. We measure LWP at midday (11.30–15.00 hours) on up to 60 plots per hour (one to three leaves per measurement) per team of five people. The flowering time and grain yield under both well-watered and stress conditions, and drought score and spikelet sterility under stress conditions are determined. These data are used to select progenitors with high drought resistance for crossing with well-adapted and accepted commercial cultivars. The progenies from these crosses are used in the routine breeding program described above.

#### Use of molecular markers

Recently, the number of lines derived from QTL-based selection has been increased in the rain-fed lowland rice breeding program. The QTL-based selection was done mostly for tolerance to disease (e.g., blast) and eating quality traits. Then, they are selected in the manner described earlier. The use of molecular-assisted selection has reduced the time to release varieties by 3–4 years, and is also more resource effective by selecting specific target traits.

#### Outcomes from the screening for drought tolerance

We have identified a number of drought-tolerant lines, e.g., three double haploid lines from a cross between CT9993 and IR62266, two lines from the rain-fed lowland rice breeding program, and seven lines from local germplasm. The double haploid lines were crossed with Surin 1 (a variety for irrigated areas), KDML105, and RD15. The latter two are popular rain-fed lowland rice varieties, and were backcrossed to BC_3_ using molecular markers, and then F_2_ materials have been selected under well-watered conditions. The Surin 1 backcross population is now undergoing field screening for drought tolerance. The populations from crosses between drought-tolerant lines and RD6 have also been developed with the aim of producing varieties with high grain yield, grain quality, and drought tolerance. These crosses have been backcrossed without using markers. The materials are used for breeding purpose as well as identifying QTLs for drought tolerance.

Already, there is some anecdotal evidence of the advantages of farmer participation in the selection of experimental lines. For example, RD12, an early maturing, blast resistant, good eating quality glutinous variety was released in early 2007 after farmer participatory selection. Adoption of this variety by the farmers is already high and increasing in northeast Thailand.

We are exploring two innovations to improve the selection process. We are determining spikelet sterility on a weight basis, weighing the total spikelets and then filled grain weight. The value is then adjusted for the difference in flowering time among lines tested. This is a quick and more accurate method. We are also improving the estimation of the time of flowering, so that we can accurately estimate delay in flowering. We are testing whether or not plot-based determination is sufficiently accurate.

### Measurement of secondary traits: Some practical considerations

*To measure flowering date*, record the date when 50% of the productive tillers in a plot have emerged. This can be a difficult date to pinpoint, especially in stressed plots where flowering is delayed, and experienced scorers can differ by as much as 3 days in their estimates of when a plot reaches 50% flowering. To improve the quality of the data, the area to be rated can be restricted to a specific central, fully bordered, part of the plot. This area will be more uniform and the data will be more consistent. Alternatively, if the crop is sown in hills, flowering date can be defined as when a certain number of hills have produced panicles. Estimates of flowering should be recorded at least three times per week.

*To measure flowering delay*, there must be an irrigated (unstressed) control treatment sown nearby. Make regular, reliable observations of flowering date to calculate the delay:

$$\begin{array}{l} \text{Floweing delay }(\text{days})=\text{days to flowering in stress treatment}\\ \;-\text{days to floweing in control treatment.} \end{array}$$

Because this character is the difference between two independent measurements of flowering date, the error is generally larger for the delay than for flowering date alone. Flowering delay is best expressed when the stress is severe, so it is easily seen in fields where drying occurs over a period of weeks. In this type of stress, lines with later flowering dates will tend to be delayed more than lines that flower early, because the stress intensity increases over time. To correct for this effect, lines can be sown with similar flowering dates in separate experiments and stress applied at the appropriate time for each experiment. Another approach is to make a statistical correction for flowering date. This can be done by using flowering date in the control as a covariate in the analysis.

*To measure spikelet fertility*, at maturity collect a sample of representative panicles from the plot. Do not use only the tallest tillers or tillers from the main stem only; these will be strongly biased. Weigh the sample. Divide the sample randomly into two, and repeat the division until the sub-sample is small enough to process. Weigh the sub-sample. Thresh the sub-sample by hand to remove all filled and unfilled spikelets. Rolling or other threshing methods cannot usually do this because, if the sample is dry, the rachis will break off with the unfilled grains or, if the sample is wet, the unfilled spikelets will remain stuck to the rachis. Separate the filled and unfilled spikelets by blowing or by flotation. Weigh the filled grains and the unfilled spikelets. Then count out 200 filled grains and record their weight, and do the same for 200 unfilled spikelets. All samples should be at the same moisture status when weighed.

$$\begin{array}{l} \text{Spikelet fertility }(\%)=(\text{number of filled grains}/(\text{number of filled}\\ \text{ grians}+\text{number of unfilled spikelets}))\times 100. \end{array}$$

where the number of filled grains is determined from the weight of filled grains in the sub sample/the mean filled grain weight and the mean filled grain weight is determined by the weight of the 200 grains sample/200.

And where the number of unfilled spikelets is determined in a similar manner to that of the filled grain.

If there are large differences in spikelet fertility among lines in an experiment, this character can be scored. Some people score in the field, but there is a tendency for scorers to look only at the tallest panicles. Other groups have found that representative panicles can be collected in the field, returned to the laboratory, and then a scorer can individually score the panicles representing each plot. The selection of panicles to harvest is critical. The sample will be more representative if all panicles from a hill are harvested.

The problem with measuring spikelet fertility is that it requires a lot of labor and, because of the many measurements required, it is prone to error. To avoid this problem, some researchers have made visual scores of percentage spikelet fertility. These scores can be used to group lines into classes of high, medium, and low fertility. Experienced scorers recommend that scoring be done on a sample of representative panicles, scoring each panicle individually, rather than trying to assign an overall plot score.

Another substitute for direct measurements of spikelet fertility is the change in the panicle harvest index (PNHI) with stress, where PNHI = grain weight/weight of panicle.

If stress has mostly affected spikelet fertility, the support structure of panicles from stress plots is similar to that of control plots, but only a proportion of the spikelets from stress plots form grains. This means that the PNHI will be lower in the stress plots. The correlation between percent fertility and PNHI is quite high for rice that experiences drought near flowering.

*To measure leaf desiccation*, make a visual integration of the symptoms in a plot, based on total leaf area lost by desiccation. A common scoring system ranges from zero (no senescence) to five (complete leaf drying). Just as for leaf rolling, it is most helpful for the final analysis if scoring is performed several times during the drought stress cycle. Because leaf desiccation is irreversible, time of day is not critical for scoring. Furthermore, since the canopy may regain turgor during the night, the morning is a good time to distinguish those parts of the canopy that are indeed desiccated and dead.

## Abbreviations

ACIAR, Australian Center for International Agricultural Research; CIAT, Centro Internacional de Agriculture; CSSL, chromosomal segment substitution lines; CV, coefficient of variation; DH, double haploid (lines); DM, dry matter; DRI, drought response index; EMS, ethyl methane sulfonate; FST, flanking sequence tags; GCP, Generation Challenge Programme; GEI, genotype-by-environment-interaction; GLD, green leaf duration; H, broad based heritability; HI, harvest index; HIF’s, heterogeneous inbred families; IRD, Institut de Recherche pour le Development (France); IRFGC, International Rice Functional Genomics Consortium; IRRI, The International Rice Research Institute; LWP, leaf water potential; LSD, least significant difference; MAGIC, multiple advanced generation intercross; MAS, marker aided selection; MET’s, multi environment trials; NIAS, National Institute of Agroecological Sciences (Japan); NERICA, new rice for Africa; OA, osmotic adjustment; PNHI, panicle harvest index; QTL’s, quantitative trait loci; RCB, randomized complete block; RGA, rapid generation advance; RIL’s, recombinant inbred lines; RNAi, RNA interference; RWC, relative water content (leaves); SAG’s, stress-associated genes; SE, selection environment; SNP, single nucleotide polymorphism; SSD, single-seed descent; T-DNA, transfer DNA-based vectors; TPE, target population of environments; WARDA, Africa Rice Center; WUE, water-use efficiency.
